# Arbuscular mycorrhizal fungi by inducing watermelon roots secretion phthalates, altering soil enzyme activity and bacterial community composition to alleviate the watermelon wilt

**DOI:** 10.1186/s12870-024-05254-7

**Published:** 2024-06-24

**Authors:** Wei Li, Chengshang Zhu, Yulu Song, Yufan Yuan, Min Li, Yingkun Sun

**Affiliations:** 1 College of Landscape Architecture and Forestry, Qingdao Agricultural University, Qingdao, 266109 Shandong P. R. China; 2 Institute of Mycorrhizal Biotechnology, Qingdao Agricultural University, Qingdao, 266109 Shandong P. R. China

**Keywords:** Arbuscular mycorrhizal fungi, Watermelon fusarium wilt, Root exudates, Phthalate, Bacterial community composition

## Abstract

**Background:**

Long-term continuous cropping has resulted in the frequent occurrence of fusarium wilt of watermelon (*Citrullus lanatus*). AMF inoculation can alleviate the continuous cropping barrier and reduce the incidence of fusarium wilt of watermelon. Our previous study found that the root exudates of mycorrhizal watermelon can enhance watermelon resistance to this disorder. It is necessary to further isolate and identify the specific compounds in root exudates of mycorrhizal watermelon and explore their control effects on fusarium wilt of continuous cropping watermelon.

**Result:**

The results of this study showed that the root system of watermelon seedlings inoculated with AMF (*Funneliformis mosseae* or *Glomus versiforme*) secreted diisooctyl phthalate (A) and dibutyl phthalate (B). Compared with water treatment, treatment with 0.1 ml/L (A1, B1), 0.5 ml/L (A2, B2) and 1 ml/L (A3, B3) of A or B significantly increased soil enzyme activities, the numbers of bacteria and actinomycetes, and the bacteria/fungi ratio in the rhizosphere. Furthermore, the Disease indexes (DI) of A1 and B3 were 25% and 20%, respectively, while the prevention and control effects (PCE) were 68.8% and 75%, respectively. In addition, diisooctyl phthalate or dibutyl phthalate increased the proportions of Gemmatimonadetes, Chloroflexi, and Acidobacteria in the rhizosphere of continuous cropping watermelon, and decreased the proportions of Proteobacteria and Firmicutes, with *Novosphingobium*, *Kaistobacter*, *Bacillus*, and *Acinetobacter* as the predominant bacteria. Compared with the water treatment, the abundance of *Neosphingosaceae*, *Kateybacterium* and *Bacillus* in the A1 group was increased by 7.33, 2.14 and 2.18 times, respectively, while that in the B2 group was increased by 60.05%, 80.24% and 1 time, respectively. In addition, exogenous diisooctyl phthalate and dibutyl phthalate were shown to promote growth parameters (vine length, stem diameter, fresh weight and dry weight) and antioxidant enzyme system activities (SOD, POD and CAT) of continuous cropping watermelon.

**Conclusion:**

Lower watermelon fusarium wilt incidence in mycorrhizal watermelons was associated with phthalate secretion in watermelons after AMF inoculation. Exogenous diisooctyl phthalate and dibutyl phthalate could alleviate the continuous cropping disorder of watermelon, reduce the incidence of fusarium wilt, and promote the growth of watermelon by increasing the enzyme activities and the proportion of beneficial bacteria in rhizosphere soil. In addition, the low concentration of phthalate diisooctyl and high concentration of phthalic acid dibutyl works best. Therefore, a certain concentration of phthalates in the soil can help alleviate continuous cropping obstacles.

**Supplementary Information:**

The online version contains supplementary material available at 10.1186/s12870-024-05254-7.

## Introduction

Watermelon (*Citrullus lanatus*) is a widely cultivated melon and cash crop, with global production exceeding 119 million tons in 2017 [1–3]. Continuous cropping of watermelon is commonly practiced because of the decreased availability of cultivated land area and the escalating demand for watermelon. However, watermelon production is limited by continuous cropping obstacles in many regions globally, causing significant economic losses [4]. Watermelon continuous cropping disorder results from multiple factors in the watermelon–soil system, including the self-toxicity of plant root exudates [5], soil salinization, and the loss of specific nutrients due to the heavy use of pesticides and fertilizers [6]. The most important factor identified is watermelon wilt caused by *Fusarium oxysporum* f. sp. niveum (Fon), which substantially harms and kills plants across all developmental stages of the crop [7]. Biological control of watermelon fusarium wilt is crucial for sustainable agriculture.

The incidence of fusarium wilt of watermelon can be significantly reduced using antagonistic microorganisms. Numerous studies have shown that arbuscular mycorrhizal fungi (AMF) can effectively control fusarium wilt [8–10]. AMF colonization can alleviate fusarium wilt of asparagus and fusarium wilt of tomato as well []. Moreover, AMF inoculation can reduce the amount of Fon in the rhizosphere of watermelon, thereby reducing the incidence of fusarium wilt of watermelon [12]. An important reason that AMF can control soil-borne diseases is that during the establishment of a symbiotic relationship with plants, AMF can induce the plant immune system to change the chemical composition of root exudates [13]. This process regulates the secretion of phytoantitoxins, phenolic acids, organic acids, and amino acids, thereby effectively killing plant pathogens [13,14]. In addition, AMF can improve soil fertility, enhance soil enzyme activity, and increase soil organic matter content [10,15].

For decades, phthalate esters (PAEs) have been widely used as plasticizers to improve the softenness and flexibility of plastics [16]. PAEs are easily released into the environment and absorbed and accumulated by the body in various ways, thus posing a significant threat to human health [17, 18]. As such, they have been regarded as major organic pollutants. PAEs are also naturally occurring substances found in bacteria, actinomycetes, fungi, ferns, higher plants, and animals [19]. They have demonstrated effectiveness in reducing soilborne diseases, improving soil properties, and promoting plant growth [20]. Dibutyl phthalate at 5 mmol.L^− 1^ significantly inhibited the proliferation of *Verticillium dahliae* Kleb in the eggplant rhizosphere [21]. In addition, dibutyl phthalate can significantly inhibit the spore germination and hyphal growth of *Colletotrichum fragariae* [22].

Root exudates play a pivotal role in the prevention and control of continuous cropping obstacles due to the inhibitory effect of certain components on soilborne pathogens [23]. Phenolic acids secreted by mycorrhizal rice and tomato roots can impede the germination of *F. oxysporum* spores [24, 25]. Coumaric acid in rice root exudates can upregulate *CIPR3* expression in watermelon root, increase β-1,3-glucanase activity in watermelon root, and enhance watermelon resistance to *F. oxysporum* [26]. Applying the root secretion of mycorrhizal watermelon to watermelon cultivated in continuous soil can effectively reduce the incidence of watermelon wilt [27]. However, the chemical composition of the root secretion of mycorrhizal watermelon associated with resistance to watermelon wilt has not been clarified. Therefore, the current study aimed to (1) isolate and identify specific compounds in root exudations of mycorrhizal watermelon and non-mycorrhizal watermelon; (2) investigate the control effect of exogenous specific compounds on watermelon wilt; and (3) evaluate the effects of exogenous specific compounds on enzyme activity and bacterial community composition in the rhizosphere soil of continuously cultivated watermelon. This study can further enrich the theoretical basis of AMF control of watermelon wilt, and provides research insights into green production and agricultural sustainable development of watermelon.

## Materials and methods

### Sample collection

In this study, Watermelon “Jingxin No. 4” was purchased from Beijing Jingyan Yinong Science and Technology Development Center. Potting soil was taken from soil that had been continuously cropped with watermelon for 10 years in Jimo, Qingdao (22°50′N, 108°17′E). The AMF *Funneliformis mosseae* and *Glomus versiforme* were provided by the Mycorrhizal Biotechnology Institute of Qingdao Agricultural University. The *F. mosseae* or *G. versiforme* inoculum consisted of a mixture of rhizospheric soil from trap cultures (Trifolium repens) containing spores, mycelium, sand, and root fragments and was sieved (< 2 mm) prior to use.

Watermelon “Jingxin No. 4"seeds were sterilized with 3% sodium hypochlorite for 10 min and washed three times using sterile water. The seeds were germinated on filter paper with sterile water in an illuminated incubator using a 12/12 h light/dark cycle at 25 °C. After three days, all uniform seedlings were transplanted into flowerpots (height × upper diameter × lower diameter = 24 cm × 24 cm × 18 cm) and cultured in a greenhouse (temperature: 25 °C, humidity: 70%, 500 µmol·m-2·s-1 light intensity, and 12/12 h light/dark cycle). And the physicochemical properties of the soil were as follows: total organic matter = 8.6 g·kg-1; available nitrogen = 11.2 mg·kg-1; available phosphorus = 19.1 mg·kg-1; exchangeable potassium = 10.3 mg·kg-1; and pH 7.10 (1:2, soil: water ratio). XAD-4 macroporous adsorption resin was purchased from Sigma-Aldrich, Schnelldorf, Germany.

### Plant material cultivation

The experiment was carried out in the greenhouse of Qingdao Agricultural University. Watermelon seeds were disinfected and germinated at 28–30℃ before seeding in plastic pots with a diameter of 16 cm containing 0.5 kg of soil. Three treatments were designed, including inoculation with *F. mosseae*, inoculation with *G. versiforme*, and the inoculation with sterilized inoculant. For inoculation with *F. mosseae* or *G. versiforme*, the growth medium was inoculated with 50 g of the inoculum (5,000 inoculant potential units) at a depth of 4 cm, and an equal amount of sterilized inoculant was added to the control [12]. Five replicates were performed for each treatment, and five watermelon seedlings were planted in a plastic pot, and the greenhouse conditions were maintained at 25/15°C day/night, 14/10 h light/dark, and relative humidity of 70% day/night. They were then cultured for 6 weeks for root exudate collection.

### Root exudates collection

After 6 weeks of inoculation treatment, all watermelon seedlings and substrates were transferred to a self-made continuous root exudate collection system (Supplementary_Material, Fig. S1), and the cultivation substrate was rinsed with enough water so that the growth of seedlings resumed in 3 days. After the circulating pump was started, the distilled water dripped into the planting basin from the top of the plant. The flow rate was maintained at 1–1.5 L·h^− 1^; the adsorption column was removed after distilled water was collected for 10 days; and the adsorption column was eluted with 500 mL of deionized water and 200 mL of *n*-hexane; there were three replicates per variety, and the eluent was added. The eluent was decompressed on a rotary evaporator at 40℃ until the *n*-hexane completely volatilized. The dry matter was dissolved using 10 mL of analytically pure methanol to obtain three types of root exudates, which were stored in a refrigerator at 4℃ for gas chromatography–mass spectrometry (GC–MS) analysis [27].

### Chemical analyses

The chemical components in root exudates were identified by GC–MS, and the experimental conditions followed recommended procedures [28]. An Agilent 6980 N gas chromatograph coupled with a mass selective detector (Agilent Technologies 5973), Agilent Chemstation software, and 30 m×0.25 mm×0.25 μm HP-5MS capillary column were used for GC–MS. The temperature of the column was initially 90 °C (1 min); it then was increased to 220 °C at 10 °C·min-1 and maintained at this temperature for 20 min. The carrier gas flow rate was 1.0 ml·min-1 (He). The sample size was 1 µl. An EI ion source was used, and the ion source temperature was 230 °C. The electron energy was 70 eV; the quadrupole temperature was 180 °C, and the full scanning range was 30–450 m·Z-1. Compounds were identified by searching the Nist standard mass spectrum library in the Agilent Chemstation chemical workstation.

### Specific compound treatment

The experiment was carried out in the solar greenhouse of Qingdao Agricultural University. Watermelon seeds were scalded and disinfected with 55 °C hot water; the watermelon plants then germinated at 28–30 °C. After germination, seeds were sown in a pot (8 × 10 × 10 cm) containing 0.5 kg of soil. Diisooctyl phthalate (A) or dibutyl phthalate (B) was purchased from Adamas Reagent Co., LTD, China. Watermelon seedlings were treated with A or B (200 ml/pot). There were seven treatments in the pot experiment: (1) A1 (0.1 ml/L A); (2) A2 (0.5 ml/L A); (3) A3 (1 ml/L A); (4) B1 (0.1 ml/L B); (2) B2 (0.5 ml/L B); (3) B3 (1 ml/L B), and (7) W (distilled water). All the pots were arranged randomly, and five replicates were performed for each treatment.

### Growth parameters

The vine length and stem diameter of watermelon were measured on days 21 after treatment using a vernier calipers, and the fresh samples of the roots and vines were placed in a constant temperature oven at 105℃ for 20 min; they were then dried to a constant weight at 80℃, and the dry weight was measured.

### Defense enzyme activity and malondialdehyde (MDA) content

Watermelon seedlings were treated for 7 d, 14 d, and 21 d, 0.5 g of normal leaves of different plants were placed into a mortar, and 0.05 mol/L phosphoric acid buffer (pH 7.8) was added; the samples were then ground in an ice bath, and the homogenate was centrifuged at -4℃ and 4,000 rpm for 20 min. The supernatant was collected to determine the activities of superoxide dismutase (SOD), peroxidase (POD), and catalase (CAT) and the content of MDA. SOD activity was determined using the nitro-blue tetrazolium (NBT) method with some modification [29], POD activity was determined using the guaiacol method with some modification [30], CAT activity was slightly modified by referring to [31]. The MDA content was quantified using the thiobarbituric acid (TBA) method [32].

### Soil enzyme activities

The activities of urease, sucrose, and CAT in the soil were measured on days 7, 14, and 21 after treatment. Urease activity was determined using sodium hypochlorite-sodium phenol colorimetry, and it was expressed as the content of amino nitrogen in 1 g of soil after 24 h. CAT activity was determined using pyrogallol colorimetry, and it was expressed as the purple gallatin content in 1 g of dry soil after 20 min. Sucrase activity was determined using 3, 5-dinitrosalicylic acid colorimetry, and it was expressed as the glucose content in 1 g of dry soil after 24 h [33].

### Microbial count in the rhizosphere soil

The number of microorganisms was measured using the dilute plate counting method [34]. The bacteria were cultured in a 37 ℃ incubator using beef extract peptone medium for 48 h, counts were then performed. The fungi were cultured on potato glucose agar (PDA) medium at 28℃ for 5 d and counted. Actinomycetes were cultured at 28℃ for 7 d using No.1 Gause medium and counted.

### Disease index (DI) and prevention and control effect (PCE)

The disease severity of watermelon plants was scored on five categories: Grade 0, normal growth; Grade 1, Leaves or vines withered from bottom to top, with the withered area accounting for 1/4 or less of the entire plant; Grade 2, Leaves or vines withered from bottom to top, with the withered area accounting for 1/4–1/2 of the entire plant, and the presence of amber colloidal substances on the vines; Grade 3, Leaves or vines withered from bottom to top, with the withered area accounting for more than 1/2 of the entire plant, and the presence of amber colloidal substances on the vines, short internodes, and the formation of a white or pink layer of mold on the surface of the lower diseased vines; and Grade 4, the entire plant is withered or dead [12].

The DI was calculated using the following formula: DI (%) = [ ∑(Ni × Ri)/(Nt × 4)] × 100% (where Ri is the disease severity scale (0, 1, 2, 3, 4), Ni is the number of leaves with a particular disease rating, and Nt is total number of leaves). The PCE was calculated using the following formula: PCE (%) = [(DI in clear water - DI in treatment)/DI in clear water] ×100% [34].

### Determination of rhizosphere bacterial community

Sample DNA was extracted using the centrifugal column soil genomic DNA extraction kit (Tiangen) per the manufacturer’s instructions. The bacterial 16 S V3-V4 region was PCR amplified using a PCR system (ABI 7500, USA) with the primer pair 341 F (5′- CCTAYGGGRBGCASCAG-3′) and 806R (5′-GGACTACHVGGGTWTCTAAT-3′) [35]. This assay uses high-fidelity polymerase to extract PCR products from bacteria. PCR amplification products were purified using the QIAGEN QIAquick PCR Purification Kit (Qiagen, Germany) per the manufacturer’s instructions.

The cDNA library was constructed using the TruSeq® DNA PCR-Free Sample Preparation Kit. The constructed cDNA library was quantified using a Qubit fluorometer and qRT-PCR; the samples were then subjected to high-throughput sequencing using an Illumina Hiseq 2500 platform (Illumina, San Diego, CA, United States). The 16 S rRNA gene sequences obtained from this study were deposited in the NCBI Sequence Read Archive database under the accession numbers SRR24008230–SRR24008250.

All paired-end raw reads were preprocessed using FLASH (Version 1.2.7, http://ccb.jhu.edu/software/FLASH/) [36]. The Clean Tags are obtained through strict filtration [37] (Lozupone,2007), and chimeric sequences were identified and removed using the UCHIME algorithm (http://www.drive5.com/usearch/manual/uchime_algo.html) to obtain the final effective tags [38] (Magoč, 2011), and all effective tags were clustered using UPARSE(Uparse v7.0.1001, http://drive5.com/uparse/) [39] (Caporaso JG, 2010), and sequences were clustered into Operational Taxonomic Units (OTUs) by default with 97% Identity. The OTUs representative sequences were annotated for species, and the RDP Classifier (Version 2.2, http://sourceforge.net/projects/rdp-classifier/) method was used for species annotation analysis with a threshold of 0.8 [40] (Edgar, 2013). Chao1, Ace, Simpson and Shannon diversity indices were calculated in QIIME, and rarefaction curves were plotted in R (Version 2.15.3). The Unweighted Unifrac distance was further calculated [41] (Whittaker, 1972). PCoA analysis and UPGMA cluster analysis were performed based on Unweighted Unifrac distance.

### Statistical analysis

The raw data were processed using Microsoft Excel (Office 2016) software (Redmond, USA). The data were analyzed using SPSS software (SPSS 16.0, Inc., Chicago, USA). The data were analyzed using analysis of variance (ANOVA). Significant differences between individual means were determined using Duncan’s Multiple Range Test (*p* < 0.05).

## Results and analysis

### Effect of AMF on root exudates of watermelon seedlings under continuous cropping

The control group, *Funneliformis mosseae*-treated group, and *Glomus versiforme*-treated group contained 15, 13, and 15 chemicals, respectively. These chemicals primarily included esters, hydrocarbons, phenols, alcohols, amines, and ketones (Supplementary_Material, Table S1).

The key chemical constituents in watermelon root exudates are esters, including linear chain esters and PAEs. In the control group, these esters in watermelon root exudates included methyl tetradecanoate, methyl palmitate, diisobutyl phthalate, and methyl cyclopentyl undecanoate (Supplementary_Material, Table S1, and Fig. S3). After *F. mosseae* inoculation, the esters in watermelon root exudates included methyl palmitate, diisobutyl phthalate, diisooctyl phthalate, and dibutyl phthalate (Supplementary_Material, Table S1, Fig. S4). After the watermelon seedlings were inoculated with *G. versiforme*, the esters present in their root exudates included methyl tetradecate, methyl palmitate, diisobutyl phthalate, dibutyl phthalate, and diisooctyl phthalate (Supplementary_Material, Table S1, Fig. S5). Both diisooctyl phthalate and dibutyl phthalate were detected in the rhizosphere soil of AMF-inoculated watermelon but not in the control, indicating their important role in the rhizosphere soil of mycorrhizal watermelon.

### Effects of phthalates on the growth of watermelon plants under continuous cropping

We treated watermelons in continuous cropping systems by using different concentrations of exogenous diisooctyl phthalate and dibutyl phthalate. The results showed that exogenous phthalate treatment could promote the growth of watermelon seedlings. Diisooctyl phthalate (A) exerted promoting the growth at low concentration and inhibiting the growth at high concentration on watermelon plant growth. Specifically, 0.5 mL/L diisooctyl phthalate treatment (A2) had the most beneficial effect on the growth and development of watermelon plants. The vine length, stem diameter, fresh weight, and dry weight of watermelons in the A2 group were 68.7%, 18.27%, 17.77%, and 62.97% higher than those in the water-treated (W) group, respectively (Table 1, Supplementary_Material, Fig. S2). Moreover, the vine length and stem diameter of the watermelons were not significantly affected by the three concentrations of dibutyl phthalate (B). However, the application of 1 mL/L dibutyl phthalate (B3) significantly increased the fresh and dry weights of the watermelon plants, which were 16.8% and 54.3% higher in the B3 group than in the W group, respectively (Table 2).

**Table 1:** Effects of diisooctyl phthalate on the growth and development of watermelon

Treatments	Vine length (cm)	Stem diameter (mm)	Fresh weight (g)	Dry weight (g)
A1	30.0b	5.65a	54.77ab	0.99ab
A2	35.6 a	5.92a	57.80a	1.14a
A3	25.7bc	5.53ab	53.87b	0.89b
W	21.1c	5.01b	49.10c	0.70c

Note: A1, A2, A3 and W indicate that watermelon was treated with 0.1 ml/L, 0.5 ml/L, and 1 ml/L diisooctyl phthalate and water, respectively. Data are mean values, and different lowercase letters indicate significant differences at *p* < 0.05. The same below

**Table 2:** Effects of dibutyl phthalate on the growth and development of watermelon

Treatments	Vine length (cm)	Stem diameter (mm)	Fresh weight (g)	Dry weight (g)
B1	27.4a	5.58a	55.27b	0.99b
B2	26.7a	5.46a	55.40ab	0.99b
B3	27.5a	5.59a	57.33a	1.08a
W	21.1b	5.01b	49.10c	0.70c

Note: B1, B2, B3 and W indicate that watermelon was treated with 0.1 ml/L, 0.5 ml/L, and 1 ml/L dibutyl phthalate and water, respectively. Data are mean values, and different lowercase letters indicate significant differences at *p* < 0.05. The same below

### Effects of phthalate esters on defense enzyme activity and MDA content in leaves of watermelon in continuous cropping systems

**Fig. 1:**
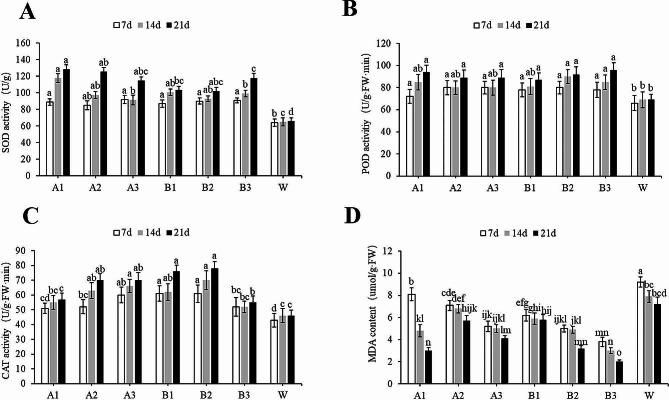
**Effects of diisooctyl phthalate and dibutyl phthalate on watermelon superoxide dismutase (SOD) activity (A), peroxidase (POD) activity (B), catalase (CAT) activity (C), and malonaldehyde (MDA) contents (D)**. Different letters above the columns indicate significant differences among means, determined by Duncan’s Multiple Range Test (*p* < 0.05)

As treatment time was extended, the use of diisooctyl phthalate as treatment significantly enhanced antioxidant enzyme activities in the leaves of watermelon in continuous cropping systems. The leaves treated with diisooctyl phthalate for 21 d exhibited significantly higher SOD activity than the leaves in the W group. The A1 group showed the highest SOD activity, which was 92.7% higher than that of the W group (Fig. 1A). The POD activity levels of the A1, A2, and A3 groups were 33.3%, 28.6%, and 28.6% higher than that of W, respectively (Fig. 1B). A higher concentration of diisoocyl phthalate indicated higher CAT activity. After 21 days of treatment, the A1, A2, and A3 groups showed CAT activity levels 17.2%, 44.8%, and 44.8% higher than that of the W group, respectively. In addition, CAT activity increased with rising diisooctyl phthalate concentration and treatment duration (Fig. 1C).

Similarly, dibutyl phthalate treatment significantly increased the activity levels of antioxidant enzymes in watermelon leaves in continuous cropping systems. After dibutyl phthalate treatment for 21 d, the leaves exhibited SOD activity significantly higher than that of the W group. Specifically, the B3 group demonstrated the highest SOD activity, followed by the B1, B2, and W groups; the SOD activity of the B3 group was 76.4% higher than that of the W group (Fig. 1A). Relative to that of the W group, the POD activity levels of the B3 groups increased by 38.1% (*p* > 0.05) (Fig. 1B). However, the B2 group treated for 21 d demonstrated CAT activity 62.0% significant higher than that of the W group (Fig. 1C).

Both diisooctyl phthalate and dibutyl phthalate inhibited malondialdehyde (MDA) synthesis in plant leaves, with MDA content decreasing as the treatment time was extended. The lowest MDA content was observed in the A1 leaves treated for 21 d, decreasing by 59.2% relative to that in the W group. However, a high concentration of dibutyl phthalate indicated greater inhibition of MDA synthesis. MDA content in B3 leaves treated for 21 d decreased by 72.2% relative to that in the W group. Therefore, low concentrations of diisooctyl phthalate and high concentrations of dibutyl phthalate exerted the most significant inhibitory effect on watermelon MDA (Fig. 1D).

### Effects of phthalate esters on enzyme activities in the rhizosphere soil of watermelon in continuous cropping systems

The activity levels of sucrase, urease, and CAT in the rhizosphere soil of watermelon increased as the duration of diisooctyl phthalate treatment was extended. In addition, high concentrations of diisooctyl phthalate (A2 and A3) more significantly promoted the activity levels of the three enzymes. The activity levels of sucrase, urease, and CAT in the A3 group treated for 21 d increased by 26.71%, 23.81%, and 31.28% relative to those in the W group, respectively. However, during the initial phase of treatment (7–14 d), no significant changes were observed in the activity levels of any of the three enzymes under any of the three concentrations of diisooctidyl phthalate (Table 3).

**Table 3:** Effects of diisooctyl phthalate on Soil enzymes

	After 7 days of treatment				After 14 days of treatment				After 21 days of treatment			
	W	A1	A2	A3	W	A1	A2	A3	W	A1	A2	A3
Sucrase	5.76b	6.63a	6.76a	6.79a	5.86b	6.72a	7.28a	7.34a	5.84c	6.86b	7.32a	7.40a
Urease	0.20b	0.23a	0.21b	0.23a	0.22b	0.25a	0.24a	0.25a	0.21b	0.28a	0.27a	0.26a
Catalase	1.70c	1.79b	1.85b	1.97a	1.72c	1.86b	1.87b	1.99a	1.79c	2.19b	2.24b	2.35a

The activity levels of sucrase, urease, and CAT in the rhizosphere soil of watermelon seedlings treated with dibutyl phthalate solution rose gradually over time. After dibutyl phthalate irrigation for 21 d, the highest sucrose activity was 26.2% higher in the B3 group than in the W group. Meanwhile, the highest urease and CAT activity levels were observed in the B1 group, which were 33.3% and 30.2% higher than that in the W group, respectively (Table 4).

**Table 4:** Effects of dibutyl phthalate on soil enzymes

	After 7 days of treatment				After 14 days of treatment			After 21 days of treatment				
	W	B1	B2	B3	W	B1	B2	B3	W	B1	B2	B3
Sucrase	5.76b	6.73a	6.91a	7.11a	5.86b	7.00a	7.09a	7.28a	5.84b	7.05b	7.33a	7.37a
Urease	0.20b	0.21ab	0.22a	0.21a	0.22b	0.23ab	0.22b	0.24a	0.21c	0.28a	0.27b	0.26b
Catalase	1.70b	1.84a	1.86a	1.82a	1.72b	1.88a	1.97a	1.85ab	1.79c	2.33a	2.26ab	2.19b

### Effects of phthalates on Fusarium wilt of watermelon under continuous cropping

Fusarium wilt was observed in watermelon seedlings across all treatment groups, with the p highest incidence (80%) reported in the W group (Table 5). Treatment of watermelon seedlings in continuously cropped soil with diisoocyl phthalate and dibutyl phthalate led decreases in the incidence, disease index (DI), and prevention and control effect (PCE) of watermelon seedlings. Within the A1 and B3 groups, the incidence was 25%, and the PCE values of A1 and B3 were 68.8% and 75%, respectively. In conclusion, a low concentration of diisooctyl phthalate and a high concentration of dibutyl phthalate can reduce the incidence and DI of watermelon wilt (Table 5).

**Table 5:** Effects of diisooctyl phthalate and dibutyl phthalate on watermelon disease status

Treatments	Disease incidence (%)	Disease indexes (DI) (%)	Prevention and Control Effect (PCE) (%)
A1	25	25	68.8
A2	50	37.5	53.1
A3	50	25	68.8
B1	50	37.5	53.1
B2	33	25	68.8
B3	25	20	75
W	80	67	16.6

### Effects of phthalates on the number of microorganisms in rhizosphere soil of watermelon in continuous cropping systems

Exogenous diisooctyl phthalate and dibutyl phthalate significantly increased the abundance of soil bacteria and actinomycetes while significantly inhibiting the abundance of soil fungi (Tables 6 and 7). The A3 and B2 groups had the most number of bacteria, 1.11 and 1.08 times that of the W group, respectively. A2 and B2 groups showed the largest numbers of actinomycetes, 2.33 and 2.26 times higher than that in the W group, respectively. The A2 and B2 groups had the least numbers of fungi, which were significantly reduced by 18.1% and 23.6% relative to that in the W group, respectively. In addition, both the A3 and B2 groups recorded the highest bacteria/fungi ratio—that is 1.53 and 1.69, respectively (Tables 6 and 7).

**Table 6:** Effects of diisooctyl phthalate on the amount of soil microbe

Treatments	Bacteria number (×10^5^CFU·g – 1 DW)		Fungi number (×10^3^CFU·g – 1 DW)	Actinomyce number (×10^3^CFU·g – 1 DW)	Bacteria / Fungi
A1	7.43b		6.15b	3.17b	1.21
A2	5.90c		5.65b	5.50a	1.04
A3	9.07a		5.95b	5.30a	1.53
W	4.30d		6.90a	1.65c	0.62

**Table 7:** Effects of dibutyl phthalate on the amount of soil microbe

Treatments	Bacteria number (×10^5^CFU·g – 1 DW)	Fungi number (×10^3^CFU·g – 1 DW)	Actinomyce number (×10^3^CFU·g – 1 DW)	Bacteria / Fungi
B1	5.63b	5.70b	5.10a	0.99
B2	8.93a	5.27b	5.45a	1.69
B3	8.33a	6.05b	3.37b	1.38
W	4.30c	6.90a	1.67c	0.62

### Effects of phthalate esters on bacterial community composition in rhizosphere soil of watermelon in continuous cropping systems

#### Sequencing quality control

After the removal of the barcodes and primer sequences, a total of 268,457 bacterial gene sequences were obtained from the soil samples. The target and chimeric sequences were then removed, retaining 200,220 bacterial gene sequences for OTU cluster analysis and subsequent analysis. The number of gene sequences obtained for each of the W, A1, A2, A3, B1, B2, and B3 groups was 29,028, 27,548, 30,101, 28,555, 25,860, 29,442, and 29,687, respectively (Table 8).

**Table 8:** Summary of sequencing data

	quality control	average length	Nontarget sequence	chimera	usable sequence
W	39,604	417	9338	1198	29,028
A1	36,989	417	8878	563	27,548
A2	40,449	416	9512	836	30,101
A3	38,268	416	8942	771	28,555
B1	34,255	416	7844	551	25,860
B2	39,081	416	9043	596	29,442
B3	39,812	417	9385	740	29,687

#### Sample complexity analysis

**Fig. 2:**
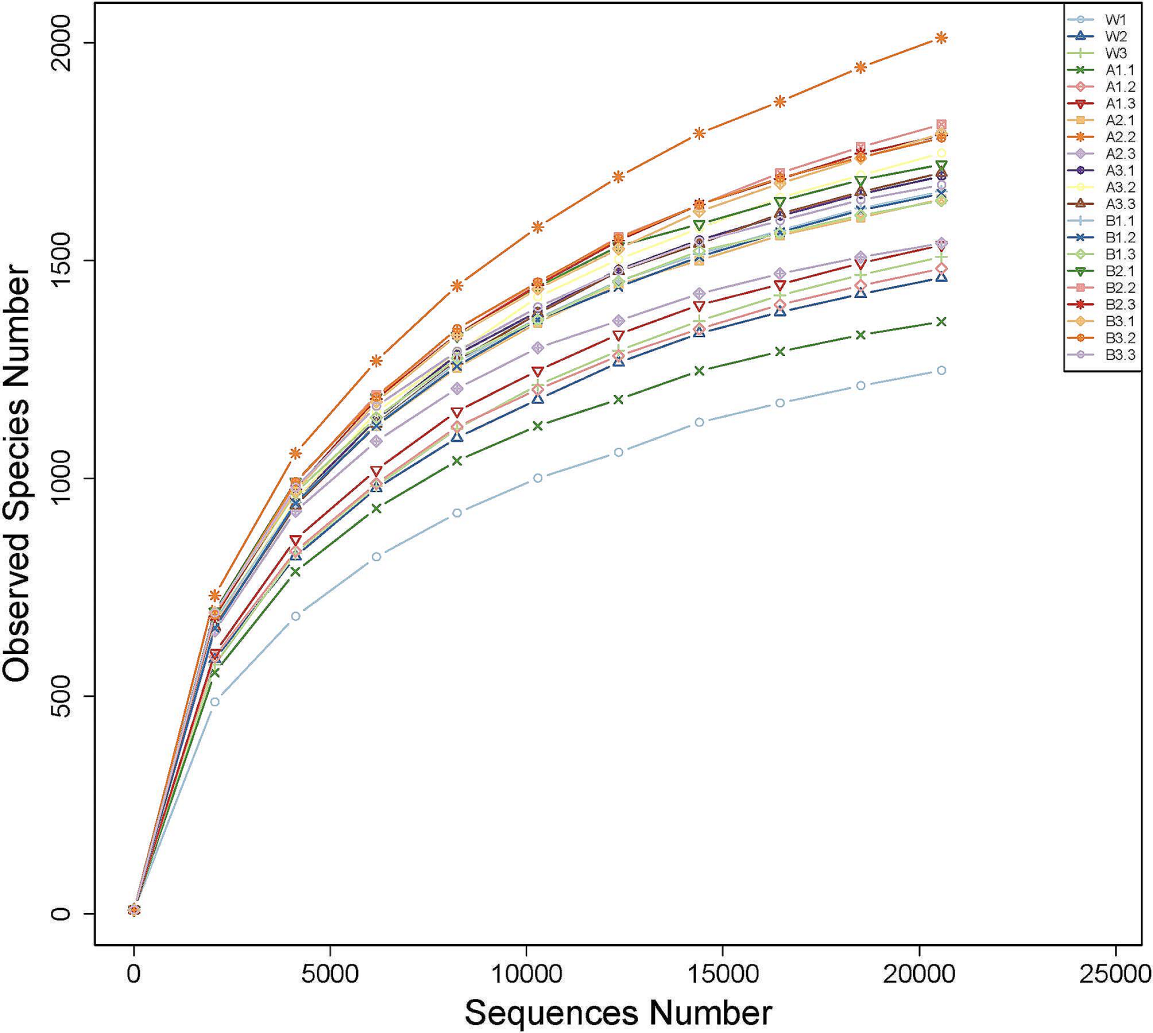
**Rarefaction curves of watermelon rhizosphere bacteria OTUs treated with diisooctyl phthalate and dibutyl phthalate at different concentrations**. A1, A2, and A3 indicate that watermelon was treated with 0.1 ml/L, 0.5 ml/L, and 1 ml/L diisooctyl phthalate, respectively. B1, B2, and B3, and W indicate that watermelon was treated with 0.1 ml/L, 0.5 ml/L, and 1 ml/L dibutyl phthalate and water, respectively, and there were three biological replicates for each treatment. The same below

Bacterial abundance was estimated using OTU abundance and alpha diversity indices including Chao1, Ace, Shannon, and Simpson indices (Table 9). Coverage exceeded 98% for all treatments, indicating the comprehensiveness of our data in portraying the species and structure of the local community (Table 9). The Shannon and Simpson indices of the A2, A3, B1, B2, and B3 groups were significantly higher than those of the W group, and the Chao1 and Ace values of the A2 and B2 groups were significantly higher than those of the W group. These findings indicate that the Chao1 and ACE values of the A2 and B2 groups were significantly higher than those of the W group (Table 9). In addition, the rarefaction curves of 21 samples from all treatment groups exhibited a gradual decrease and flattening but did not reach saturation. These observations suggest that the sequencing data were robust and that the database sample size could better reflect the diversity of microorganisms in the environment. Collecting more data may only yield a small number of new species (OTUs) (Fig. 2).

**Table 9:** Alpha diversities in different treatments

Treatments	Shannon	Simpson	Chao1	ACE	Goods coverage
A1	8.16b	0.9833b	1660.86b	1722.59ab	0.9833
A2	8.92a	0.9940a	2048.04a	2071.95a	0.9797
A3	8.85a	0.9943a	1953.10ab	2019.50ab	0.9803
B1	8.87a	0.9943a	1865.99ab	1887.78ab	0.9830
B2	8.88a	0.9937a	2087.68a	2096.85a	0.9793
B3	8.95a	0.9943a	2057.89a	2065.11ab	0.9797
W	8.16bB	0.9860bAB	1630.76bA	1691.52bA	0.9830

#### OTU analysis and species

**Fig. 3:**
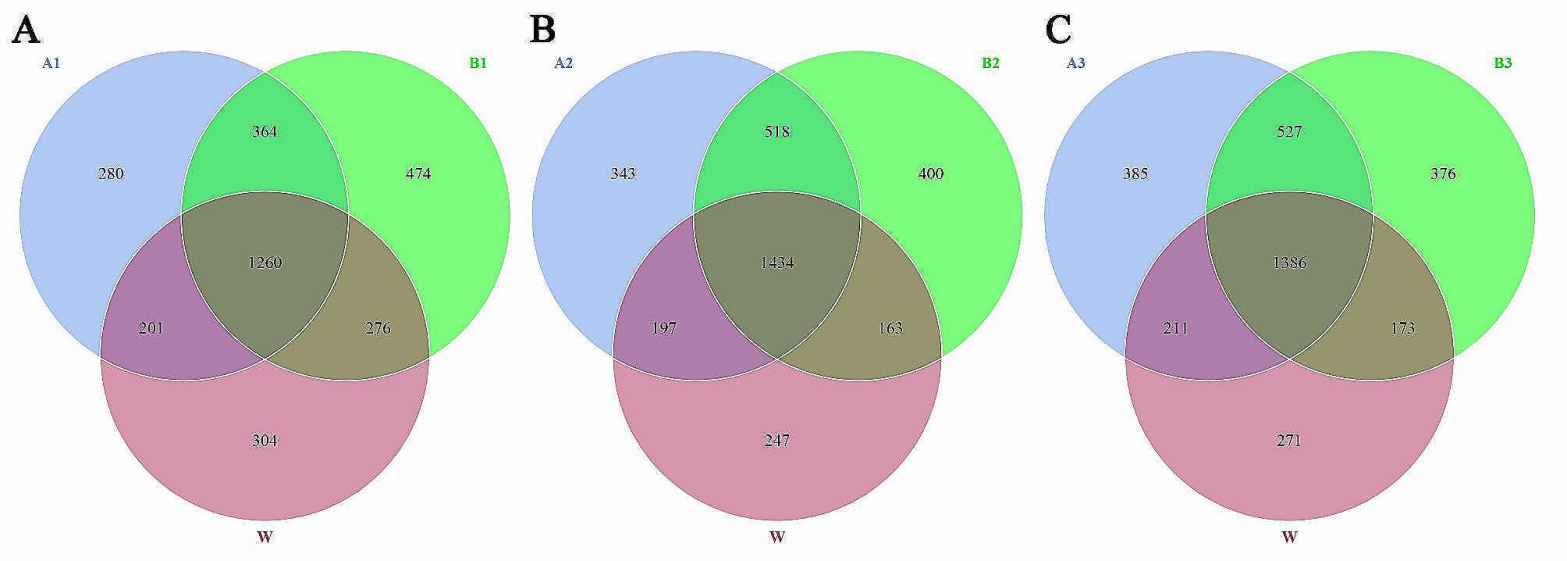
Venn diagram of microbial communities following treatment of soil with different concentrations of diisooctyl phthalate and dibutyl phthalate

The common and unique OTUs among different treatments were analyzed based on the results of OTU cluster analysis, and a Venn diagram was generated. Figure 3 presents a comparison among groups treated with the same concentration of exogenous diisooctyl phthalate or dibutyl phthalate. Each circle in the figure represents a treatment, and the overlap of the circles represents the number of OTUs shared between treatments. Watermelon seedlings in the A1 or B1 groups had 3,159 unique bacterial OTUs. Of this number, A1 shared 201 OTUs with W; B1 shared 276 OTUs with W; A1 shared 364 OTUs with B1; and A1, B1, and W shared 1,260 OTUs (Fig. 3A). When watermelon seedlings in the A2 or B2 groups had 3,302 unique bacterial OTUs. Of this number, A2 shared 197 OTUs with W; B2 shared 163 OTUs with W; A2 shared 518 OTUs with B2; and A2, B2, and W shared 1,434 OTUs (Fig. 3B). And watermelon seedling in the A2 or B2 groups had 3,329 unique bacterial OTUs. Of this number, A3 shared 211 OTUs with W; B3 shared 173 OTUs with W; A3 shared 527 OTUs with B3; and A3, B3, and W shared 1,386 OTUs (Fig. 3C). Therefore, when the same concentration of exogenous diisoocyl phthalate or dibutyl phthalate was used for the treatment of watermelon seedlings in continuous cropping systems, the similarity between each treatment and W exceeded 69%. The similarity between A2 and B2 reached 0.78, and that between A3 and B3 was 0.77 (Table 10).

**Table 10:** Comparison of the degree of similarity between each sample in the horizontal direction

	A1	A2	A3	B1	B2	B3	W
A1	1						
A2		1					
A3			1				
B1	0.73			1			
B2		0.78			1		
B3			0.77			1	
W	0.70	0.72	0.70	0.70	0.70	0.69	1

#### Relative abundance analysis of species

**Fig. 4:**
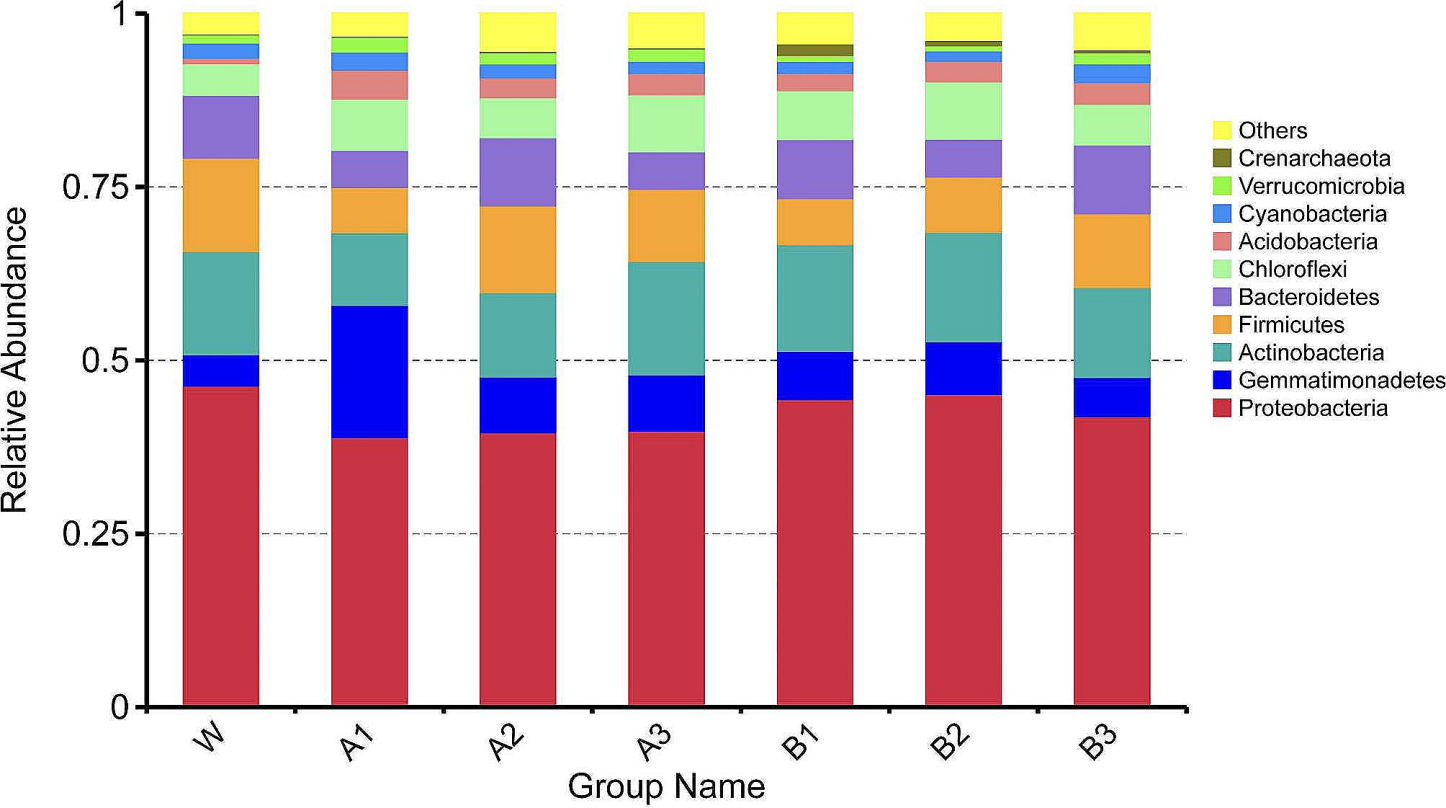
Effects of diisooctyl phthalate and dibutyl phthalate on watermelon rhizosphere soil bacterial community at the phylum level

Significant changes in the predominant bacterial species in the rhizosphere soil of continuously cultivated watermelon were observed across different treatment groups. Proteobacteria was identified as the primary dominant phylum in the rhizosphere soil of watermelon under continuous cropping, comprising more than 38.92% of the total bacterial population. Exogenous diisooctyl phthalate or dibutyl phthalate increased the abundance of Gemmatimonadetes, Chloroflexi, and Acidobacteria, while decreased the abundance of Proteobacteria and Firmicutes in the rhizosphere of watermelon in continuous cropping systems. Compared with that in the W group, the abundance of Gemmatimonadetes increased by 3.22 times in the A1 group and by 68.18% in the B2 group. In addition, the abundance of Acidobacteria increased by 4.20 times in the A1 group and by 2.68 times in the B2 group. Moreover, the abundances of Proteobacteria and Firmicutes in the A1 group were 16.08% and 51.49% lower than those in the W group, respectively. The abundances of Proteobacteria and Firmicutes in the B1 group were 4.17% and 50.84% lower than those in the W group, respectively (Fig. 4).

**Fig. 5:**
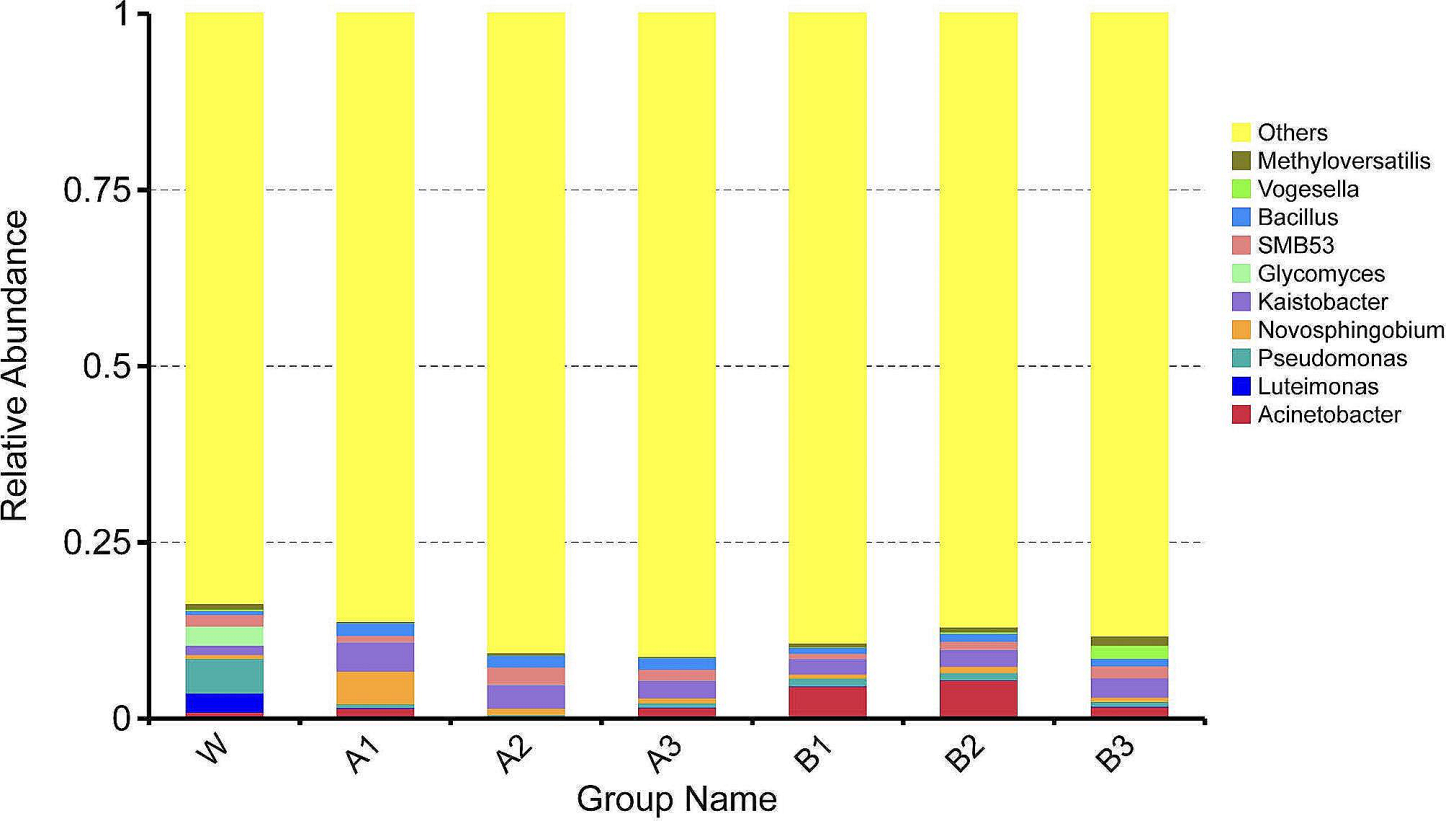
Effects of diisooctyl phthalate and dibutyl phthalate on watermelon rhizosphere soil bacterial community at the genus level

**Fig. 6:**
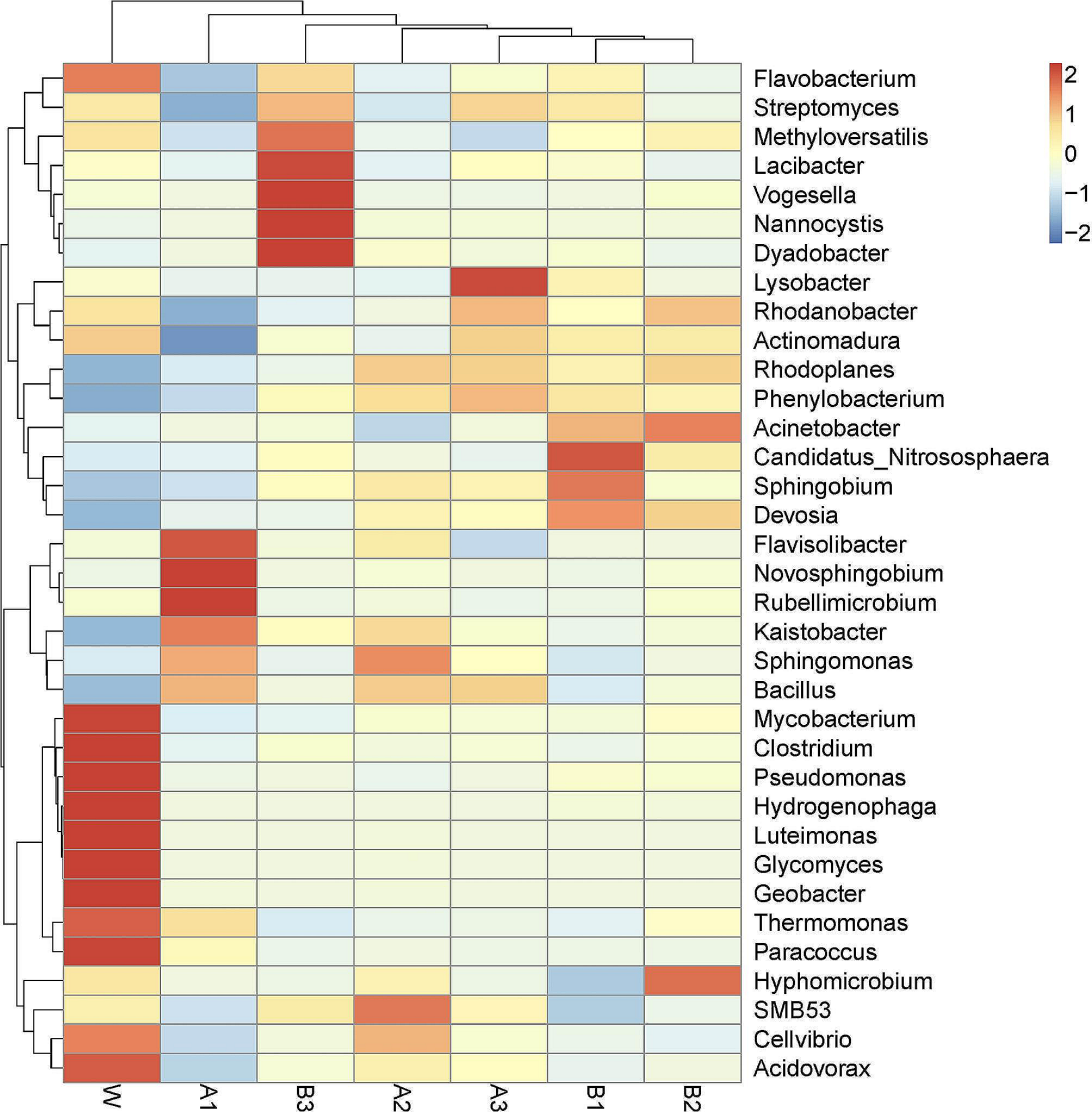
**Heatmaps and dendrograms of bacteria based on the relative abundance of dominant genera rhizosphere bacteria in watermelon treated with diisooctyl phthalate and dibutyl phthalate at different concentrations.** The heatmap plot indicates the relative abundances of genera in different soil samples. The color intensities are proportional to the relative abundances of the genera. The phylogenetic tree was calculated using the neighbor-joining method

The predominant genera identified in the W group were *Pseudomonas*, *Glycomyces*, and *Luteimonas*. After the watermelon seedlings in continuous cropping systems were treated with exogenous diisooctyl phthalate and dibutyl phthalate treatment, the predominant genera were *Novosphingobium*, *Kaistobacter*, *Bacillus*, and *Acinetobacter*. Following the treatment of watermelon seedlings in continuous cropping systems with different concentrations of exogenous diisooctyl phthalate and dibutyl phthalate, the abundance of *Pseudomonas* was reduced by 91.92%, and that of *Saccharomyces* decreased by 98.20% in the A2 group relative to the W group. Meanwhile, the abundance of *Pseudomonas* was reduced by 86.37%, and that of *Saccharomyces* decreased by 99.70% in the B3 group. In addition, the abundances of *Novosphingobium*, *Kaistobacter*, and *Bacillus* in the A1 group were 7.33, 2.14, and 2.18 times higher than those in the W group, respectively; meanwhile, those in the B2 group were 60.05%, 80.24%, and 1 time higher than those in the W group, respectively (Figs. 5 and 6).

#### Comparative analysis of multiple samples

**Fig. 7:**
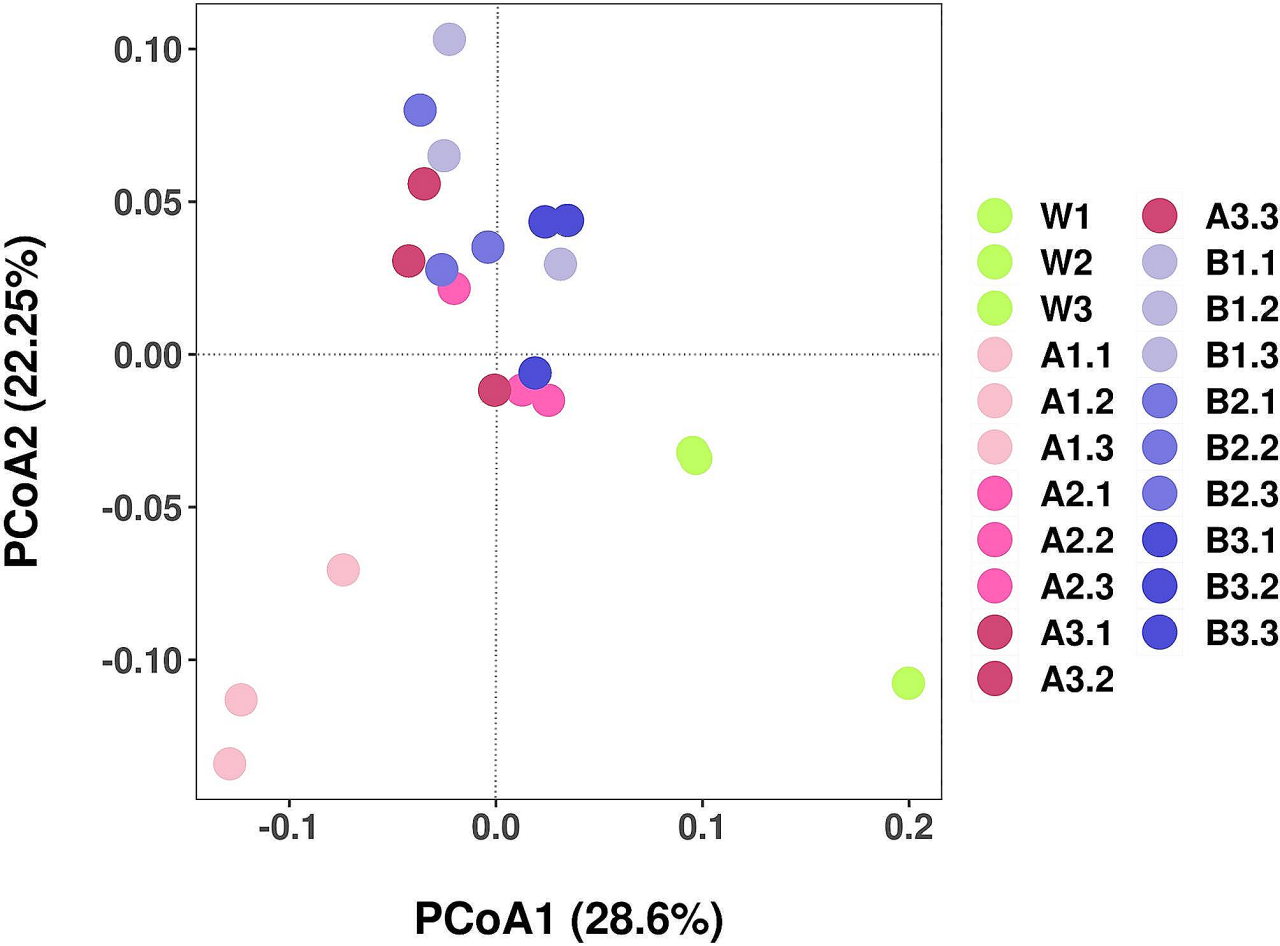
Principal coordinate analysis of bacterial community following treatment of soil with different concentrations of diisooctyl phthalate and dibutyl phthalate

Principal coordinate analysis (PCoA) can be used to characterize the similarities and differences among data sets. It is an unconstrained method for reducing data dimensionality that can be used to investigate the similarities and differences in community composition. The results of PCoA, based on weighted UniFrac distance and bacterial β-diversity analysis, indicated a discernible distance between rhizosphere soil samples in various treatment groups. The contribution rate of PCoA1 was 28.6%, and that of PCoA2 was 22.25%, resulting in a cumulative contribution rate of 50.85% for PCoA1 and PCoA2 combined. Each treatment group was relatively clustered, with the A1–A3 groups and B1–B3 groups distant from the W group, indicating that the observed change in β-diversity occurred following the treatments with diisooctyl phthalate and dibutyl phthalate treatments, while the water treatment only slightly influenced the bacterial community composition in the rhizosphere soil of continuously cropped fields. In addition, the relatively substantial distance between the A1 group and the other treatment groups suggests that soil bacterial community composition exhibited greater sensitivity to to low concentrations of diisooctyl phthalate (Fig. 7).

**Fig. 8:**
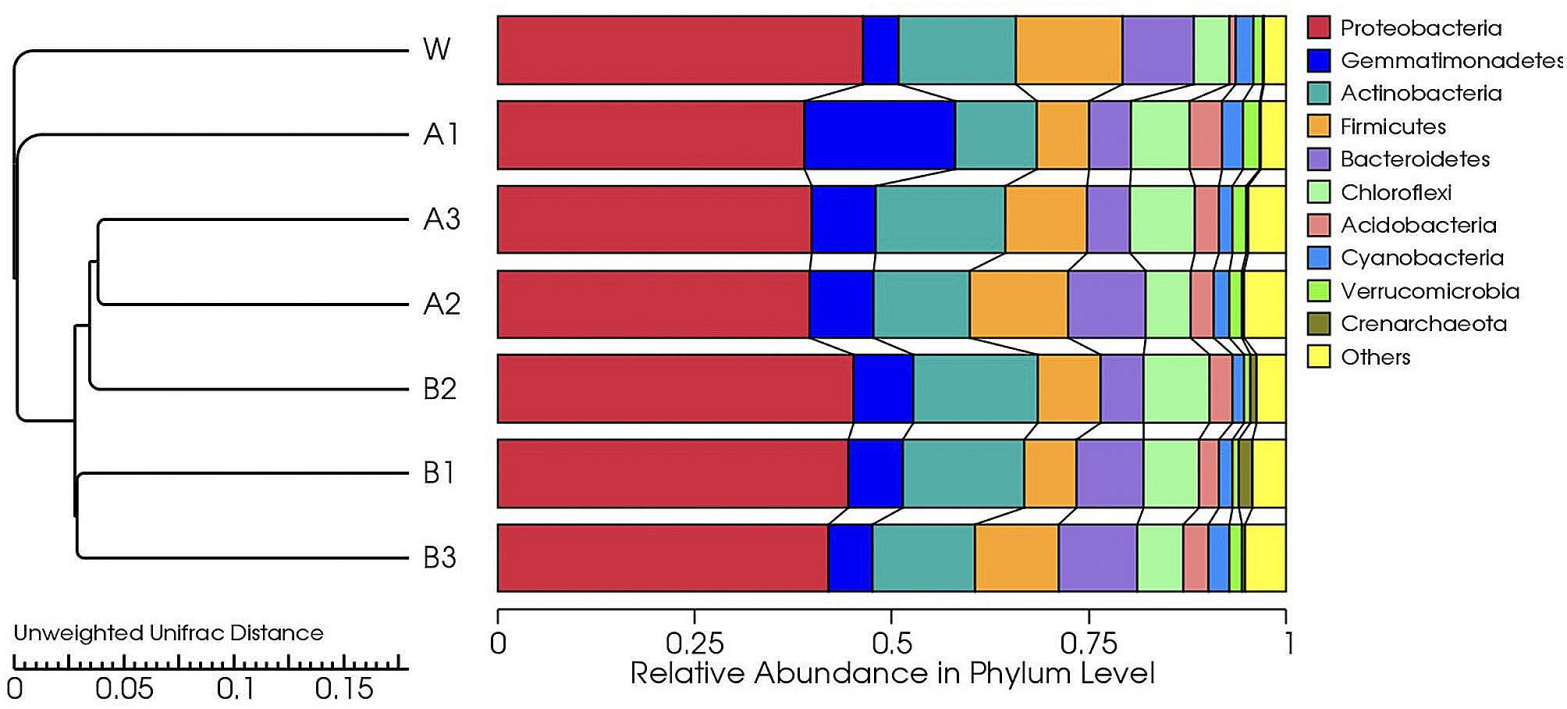
Cluster analysis of the dominant genera in different soil samples based on unweighted UniFrac distances

The clustering tree showed that all samples were grouped into two distinct clusters overall (Fig. 8). The W group formed a separate branch, whereas the remaining treatment groups were clustered into another branch. In addition, the A1 group was distant from the other treatment groups; meanwhile, the A2, A3, and B2 formed a cluster, and the B1 and B3 groups formed another. In conclusion, both exogenous diisooctyl phthalate and dibutyl phthalate treatments induced changes in the bacterial community composition of watermelon rhizosphere soil, with the A1 treatment group exhibiting the most significant change (Fig. 8).

## Discussion

Continuous cropping obstacles substantially affect watermelon yield and quality [4] and disrupt the composition of soil microbial communities. Long-term continuous cropping significantly alters the diversity of bacteria and fungi within the watermelon rhizosphere [42]. A primary contributing factor to watermelon continuous cropping disorder is the capacity of watermelon root exudates to stimulate Fon sporulation and spore germination [43]. The inoculation of exogenous microorganisms can alter the composition of root exudates in host plants, playing a crucial role in conferring resistance to watermelon continuous cropping disorder. Previous studies have demonstrated that AMF inoculation can regulate the composition of watermelon root exudates, increasing the secretion of P-coumaric acid and malic acid. This mechanism leads to a reduction in the disease index of *Fusarium* wilt of watermelon by 89.3% and mitigates watermelon continuous cropping disorder [12]. In our previous study, we directly used root exudates from mycorrhizal watermelon to treat watermelons cultivated under continuous cropping conditions, resulting in a significant reduction in the incidence of Fusarium wilt [27]. Considering this finding, we further investigated the specific role of individual components within the root exudates of mycorrhizal watermelon in the control of Fusarium wilt of watermelon.

In the current study, the root exudate profiles of mycorrhizal and nonmycorrhizal watermelon seedlings were determined by gas chromatography-mass spectrometry (GC-MS) using soil from 10 years of continuous cropping. Diisooctyl phthalate and dibutyl phthalate were detected in the root exudates of mycorrhizal watermelon; by contrast, they were absent in the C1, C2, and W groups. We hypothesize that these two compounds may contribute to the decreased incidence of Fusarium wilt in mycorrhizal watermelon. Therefore, exogenous diisooctyl phthalate and dibutyl phthalate were applied to watermelon cultivated in continuously cropped soil. Compared with those of the control group, the watermelon growth parameters and the SOD, POD, and CAT activities improved in the groups treated with he dibutyl phthalate and diisooctyl phthalate. The control effect of these compoundsFusarium wilt of watermelon could reach 68.8–72%. Previous studies have confirmed that some PAEs found in root exudates of various crops can effectively reduce soilborne diseases, improve soil properties, and promote plant growth [20, 44, 45]. For instance, dibutyl phthalate and diisobutyl phthalate inhibited *Meloidogyne incognita* J2 [45], dibutyl phthalate significantly inhibited spore germination and hyphal growth of *C. fragariae* [22], 5 mmol·L^-1^ dibutyl phthalate significantly suppressed the proliferation of *V. dahliae* Kleb in the rhizosphere of eggplant [21]. The results of current study further confirmed that different phthalates exert common control effects on various soilborne pathogens, but the inhibition of exogenous phthalates depends on the applied concentration [26].

Soil enzymes represent one of the most dynamic components within soil, mainly originating from the exudates of soil microorganisms and plant roots. They directly participate in crucial biochemical processes of soil, and their activities serve as key indicators for evaluating soil quality [46]. Previous indications suggested that dibutyl phthalate could increase soil CAT activity while decreasing soil urease activity [47–51]. PAEs have also been reported to inhibit soil dehydrogenase and urease activities [49, 51, 52]. In the present study, both exogenous diisooctyl phthalate and dibutyl phthalate could increase the activities of urease, sucrase, and catalase in the rhizosphere of watermelon in continuous cropping systems. This occurrence might be linked with environmental factors, soil types, PAE types, and PAE concentrations.

Exogenous phthalate treatment could alter the species and quantity of microbial communities in the root soil of host plants. Studies have shown that PAEs can reduce soil bacterial diversity [53]. Wang et al. (2017) found that di(2-ethylhexyl) phthalate (DEHP) could affect the evenness, Shannon index, and richness index of soil microorganisms [54]. Moreover, DEHP could enrich microorganisms with the ability to degrade phthalate, such as *Rhizobium* and *Agromyces* [55–57]. The results of the current study indicate that diisoprooctyl phthalate and dibutyl phthalate increased the abundance of bacteria and actinomycetes in rhizosphere soil and decreased the abundance of fungi. The bacteria-to-fungi ratio in the rhizosphere soil of the A and B groups exceeded that of the W group, suggesting a shift in the soil microbial population from low-fertility “fungal” soil to high-fertility “bacterial” soil in the A and B groups, promoting the growth of watermelon seedlings. Earlier studies have also confirmed that an increase in the copy number and bacterial diversity of actinomycetes could reduce the incidence index of Fusarium wilt of strawberry [10]. The current study also obtained essentially consistent results, indicating that the exogenous application of diisooctyl phthalate and dibutyl phthalate could also reduce the incidence and DI of watermelon wilt.

Phthalate production may occur commonly both on land and in the sea. Some PAEs have been found in root exudates of various crops, effectively reducing soilborne diseases, improving soil properties, and promoting plant growth [20, 44, 45]. Rice roots can secrete p-coumaric acid, salicylic acid, and phthalic acid, exerting a potent inhibitory effect on spore germination and sporulation of Fon [43]. Dibutyl phthalate secreted by leek demonstrates effective control over Fusarium wilt of *Momordica charantia* [58]. The results of present study were consistent with previous findings. Exogenous application of diisooctyl phthalate and dibutyl phthalate also led to reductions in the incidence and DI of Fusarium wilt of watermelon. In addition, exogenous phthalates could alter the composition of the soil microbial community. When the concentration of dibutyl phthalate exceeded 50 mg·L − 1, the relative abundance of Firmicutes increased, whereas that of Bacteroidetes decreased [59]. In the present study, the abundances of *Bacillus* and Acidobacteria in the rhizosphere soil of watermelon were the highest in the A1 group; those of *Actinomyces* and *Aspergens* were the highest in the B2 group; and those of *Bacteroides* were the highest in the B3 group. Acinetobacter calcoaceticus exhibits antagonism toward various plant pathogens such as *F. oxysporum*, *Aspergillus flavus*, and *Acinetobacter nigricans* [60]. Krishnaraj reported that Acinetobacter can control Fusarium wilt of tomato and improve plant growth under greenhouse conditions [61]. *Bacillus amylolitica* DHA55 inhibited Fon in watermelon roots [62]. In conclusion, both phthalates in the present study can enhance bacterial community diversity and render the rhizosphere environment more conducive to plant growth by recruiting beneficial microorganisms or inhibiting pathogenic microorganisms [63]. Meanwhile, beneficial microorganisms may resist soilborne diseases by upregulating the expression of defense-related genes [64] or strengthening root cell wall structures to prevent pathogens from directly penetrating plant roots [52]. Regardless, PAEs persist as organic pollutants in agricultural soils [65], requiring prudent caution in their application for soilborne disease control to avert the potential for significant organic pollution.

## Conclusion

In the present study, *Funneliformis moseae* or *Glomus versiformme* were used as inoculants to induce the secretion of diisooctyl phthalate and dibutyl phthalate from watermelon, which significantly reduced the incidence and DI of fusarium wilt of watermelon. This reduction was attributed to the following: heightened activity levels of plant antioxidant enzymes; enhanced soil enzyme activities; induction of fungal-to-bacterial soil transformation; elevated abundance of Gemmatimonas, Chloroflexi, and Acidobacteria; and reduced abundance of Proteobacteria and Firmicutes. In addition, phthalates significantly improved the growth (vine length, stem diameter, fresh weight, and dry weight) and defense enzyme activities (SOD, POD, CAT) of continuous cultivation watermelon.

### Electronic supplementary material

Supplementary Material 1

## Data Availability

All data generated or analysed during this study are included in this published article [and its supplementary information files].
